# Puerarin-coated gold nanoparticles (PUE-AuNPs) synthesized via green synthetic route: a new colorimetric probe for the detection of ciprofloxacin

**DOI:** 10.3906/kim-2106-4

**Published:** 2021-07-21

**Authors:** Tasneem ZEHRA, Faiqa AHSAN, Muhammad Ali VERSIANI, Sana WAHID, Sajid JAHANGIR, Muhammad Raza SHAH

**Affiliations:** 1 Department of Chemistry, Federal Urdu University of Arts, Science & Technology, Karachi, Pakistan; 2 Basic Science, Mathematics, English & Humanities Department, Dawood University of Engineering and Technology, Karachi, Pakistan; 3 H.E.J. Research Institute of Chemistry, International Center of Chemical and Biological Sciences, University of Karachi, Karachi, Pakistan

**Keywords:** Gold nanoparticles, puerarin, ciprofloxacin, colorimetric method, sensor

## Abstract

Puerarin-coated gold nanoparticles (PUE-AuNPs) synthesized via green synthetic route, a new colorimetric sensor, efficiently detected the ciprofloxacin (CP) in tap water and cow milk samples. The PUE-AuNPs were characterized by UV-visible, FTIR, AFM, and DLS techniques and were found to be spherical with an average size of approximately 19–20 nm. FTIR spectrum confirms that functional groups such as −OH, −C=O, −CO and −C=C were responsible for the reduction of gold (III) chloride trihydrate. These functional groups acted as capping agents to form AuNPs. The PUE-AuNPs sensor was proved to be selective and sensitive for the detection of CP through colorimetric method within the concentration of 1 to 1000 *μ*M and the limit of detection was 51 *μ*M. This colorimetric sensor is simple, cost-effective, and selective towards CP detection in environmental (tap water and milk) samples.

## 1. Introduction

Antibiotics are the group of medicines which not only ameliorate the human health but their applications have also enhanced veterinary medicine as well as agriculture and farming [1]. However, due to the large consumption of antibiotics, they reach the sewage treatment plants through excretion process, where they are not completely removed and easily enter into the natural water courses (probably drinking water), soil, milk, and meat samples, which is hazardous for both human and animal health [2,3]. Due to their irregular entering into the environment and enduring presence, this category of pharmaceuticals is considered to be persistent and “pseudopersistent” contaminants [1]. Antibiotic use is estimated to be between 100,000 and 200,000 t per year worldwide. Untargeted species are adversely affected by antibiotic contamination in the aquatic ecosystem like preventing algae growth, facilitating bacterial resistance, and damaging chloroplast replication, interrupting the microorganism *N* cycle, translation, and transcription [4].

**Figure 1:**
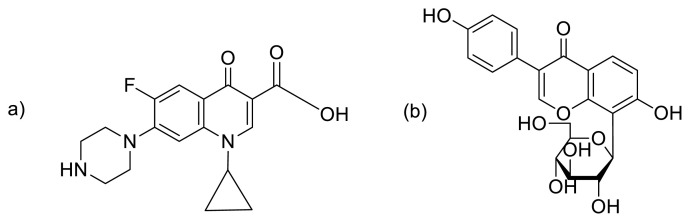
(a) Structure of ciprofloxacin and (b) puerarin.

Fluoroquinolones is one of the most important classes of second-generation antibiotics. Administered fluoroquinolones are mostly expelled as unchanged compounds in urine and are subsequently discharged into hospital or municipal sewage. These fluoroquinolones are not completely removed at waste water treatment process (WWTPs), and subsequently, their persistent presentation into the environment makes fluoroquinolones ‘pseudopersistent’ compounds [5]. Moreover, their potential to promote antibiotic resistance, fluoroquinolones also have an unfavorable ecotoxicity profile, and may contribute to a significant portion of the measured bacterial genotoxicity in hospital effluents. CP [1-cyclopropyl-6-fluoro-4-oxo-7-piperazin-1-ylquinoline-3-carboxylic acid] (Figure 1a) is 65% excreted in urine and only 25% in the feces. It is the most widely prescribed antibiotic which is active against a broad spectrum of gram-negative and gram-positive bacteria [6], and has strong bioavailability, tissue penetration, minimal side effects, and desirable biological fluid distribution characteristics [7]. It grasps the aqueous atmosphere through various ways, like household wastewater, chemical pollution, municipal waste collection, manure from animals, and garbage dumps. The excess of CP also cause some severe diseases including nausea, vomiting, diarrhea, and abdominal discomfort [8], agranulocytosis (reduced white blood cells), and toxic epidermal necrolysis (severe skin reaction resulting sepsis or death) [9], and neurological side effects like abnormal vision, acute organic psychosis, and seizure [10].

CP was consistently observed in a number of water environments due to these various entry routes [11]. In Switzerland, CP was detected in the range 249–405 ng/L and 45–568 ng/L in domestic sewage and at WWTPs respectively, 0.6–2 μg/L and 0.02 μg/L of CP was also detected in wastewaters and surface streams across the US. Moreover, CP in the range 0.7–124.5 μg/L was found in wastewater of a Swiss hospital [6]. Due to the aforementioned concerns, various methods have been employed so far, to detect the ciprofloxacin in many environmental samples, such as fluorescence spectroscopy [12], cyclic voltammetry [13], high-performance liquid chromatography with fluorescence [14], *β*-galactosidase-based colorimetric assay [15], amperometric, and electrophoresis with conductivity analysis [16], and liquid chromatography–mass spectrometry method [17] (Table 1). Although these approaches offer sensitive and precise multianalytical detection, they also require a lot of time, costly instruments, tricky sampling, and utilization of detrimental natural solvents in massive portions as well as particularly skilled professional operators. Therefore, there is a dire need to develop a simpler, quicker, and more reliable method for the detection of CP in environmental samples. Spectrophotometric-based colorimetric detection methods have many benefits such as simple use, high sensitivity, high reliability, and being inexpensive. In the present study, we account a green synthetic route to synthesize gold nanoparticles (AuNPs) for the first time using puerarin (PUE) (an isoflavonoid, isolated from *Pseudocalymma elegans*) as a reducing and stabilizing agent. The morphology of the PUE-AuNPs was characterized by atomic force microscopy (AFM) and scanning electron microscopy (SEM). Puerarin **[**8-(*β*-D-glucopyranosyl-7-hydroxy-3-(4-hydroxyphenyl)-4*H*-1-benzopyran-4-one**]** is a white powder and is soluble in methanol (Figure 1b) [18]. It possesses several pharmacological properties such as antiinflammatory, anticancer, antioxidant, antidiabetic, and cardiac- and neuro-protective. Previously, puerarin-coated nanoparticles were synthesized through different methods like anionic polymerization [19], emulsion solvent evaporation [20], solvent evaporation [21], and precipitation [22]. We have planned a cost-effective, simple, and fast colorimetric chemosensing platform for the detection of CP. The developed colorimetric method is based on the vigorous interaction among the Au and NH/OH group of CP and thus forming Au-N/O bond. The CP binds on the surface of PUE-AuNPs, which results in a high degree of aggregation as confirmed by UV-visible and FTIR spectroscopy, as well as AFM and DLS analysis. The proposed method described here is practically applicable for the detection of CP in environmental (tap water and milk) samples. The sensor was proved to be selective and sensitive for the detection of CP in presence of other drugs.

## 2. Experimental

### 2.1. Materials and instruments

Gold chloride [H(AuCl_4_).3H_2_O] (Korea/China) and methanol (Merck) were purchased from the market, Puerarin was isolated from *Pseudocalymma elegans* (vide Section 2.2). Generic ciprofloxacin amoxicillin trihydrate, azithromycin, cefotaxime, cephradine, diclofenac sodium, esomeprazole, flurbiprofen, levetiracetam, levofloxacin, mefenamic acid, metronidazole, omeprazole, paracetamol, penicillin G. procaine, piroxicam beta cyclodextrin, and zuclopenthixol were obtained from pharmaceutical industries. Glassware was cleaned with aqua regia to avoid possible contamination with metals and washed with deionized water. A UV-visible double beam spectrophotometer (Shimadzu, CE 7200, 190–900 nm) was used for UV-visible spectra imaging. A Brucker vector spectrometer was used for FTIR spectroscopy in IR range 500–4000 cm^−1^. A zeta-sizer, Nano-ZSP (Malvern Instruments) was used before and after drug interaction to test particle size distribution and zeta potential of nanoparticles. Measurements were conducted at a scattering angle of 90° at 25 °C. A quartz cuvette was used to evaluate the particle size while zeta potential was established in the dip cell cuvette. Surface morphology and 3D view of PUE-AuNPs was viewed on an atomic force microscope (AFM, Agilent 5500, Arizona, USA) prior to and after drug interaction. The preparation of sample was carried out by putting a drop of analyte into the silicon wafer substrate and dried for almost 24 h for analysis. A triangular soft silicon nitride cantilever (Veeco, template MLCT-AUHW) with a spring constant of 0.1 Nm^−1^ and a marginal value of 0.01 Nm^−1^ was used in tapping mode for the measurements. Scanning electron microscope (SEM) coupled with energy dispersive spectroscopy (EDX) was done using SEM JEOL JSM-6380A.

### 2.2. Isolation of puerarin

Freshly collected young leaves (5 kg) of *Pseudocalymma elegans* were extracted in methanol, and the obtained methanolic extract (PELM, 500 g) was partitioned between aqueous (PELMW, 350.0 g) and ethyl acetate (PELME, 150.0 g) phases. The aqueous phase was lyophilized, and the obtained concentrated residue PELMW (200 g) was subfractionated from nonpolar to polar solvent. Puerarin was obtained from butanol soluble fraction (PELMWB, 1.0 g) through normal pressure column chromatography [23].

### 2.3. Synthesis of PUE-AuNPs

For PUE-AuNPs synthesis, 14 mL of HAuCl_4_.3H_2_O (1mM) solution was diluted with 180 mL of distilled water into a beaker, and the solution was heated up to the boiling point. After that, 1.5 mL of 1 mM of puerarin (PUE) was poured into a flask. The mixture changed from colorless to light pink after the addition of PUE solution within a few minutes. The mixture of Au and PUE was heated until the solution turned to pink or red. The shift in color confirmed the formation of puerarin-coated AuNPs. After the formation of puerarin-coated AuNPs, the pH of the solution was found to be 5.8.

### 2.4. Recognition of CP and its mechanism

The PUE-AuNPs chemosensing ability against several drugs (such as amoxicillin trihydrate, azithromycin, cefotaxime, cephradine, ciprofloxacin, diclofenac sodium, esomeprazole, flurbiprofen, levetiracetam, levofloxacin, mefenamic acid, metronidazole, omeprazole, paracetamol, penicillin G. procaine, piroxicam beta cyclodextrin, and zuclopenthixol) was analyzed. To study the potential drug sensing, drug solutions were taken in Eppendorf tubes in 1:1(v/v) ratio with PUE-AuNPs. Briefly, 1 mL of 1 mM of different drugs were added individually into 1 mL of PUE-AuNPs and incubated for 15 min at room temperature (28 ± 2 °C). Initially, the feasible mode of interaction (clustering or aggregation) between drugs and PUE-AuNPs was studied through UV-visible spectroscopy. The confirmation of the molar ratio of interaction between CP and PUE-AuNPs was studied by plotting Job’s plot.

### 2.5. Preparation of environmental samples

The tap water was collected from the Department of Chemistry, Federal Urdu University of Arts, Science, and Technology, Karachi, Pakistan and cow milk was purchased from a local milk shop in Karachi, Pakistan. The samples were used without additional treatment. Three solutions **A**, **B**, and **C** were prepared for tap water analysis. The solution volume was set at 3 mL. Sample **A** contained 2 mL of tap water and 1 mM of ciprofloxacin 2:1 ratio, **B** contained tap water and 1 mM of PUE-AuNPs 2:1 ratio while **C** contained tap water, 1 mM of PUE-AuNPs and 1 mM of ciprofloxacin 1:1:1 ratio, and they were incubated for 15 min at room temperature (28 ± 2 °C) then screened through UV-visible spectroscopy. A similar practice was followed for the examination of CP in the cow milk sample [24].

## 3. Results and discussion

### 3.1. Characterization of PUE-AuNPs

**Figure 2:**
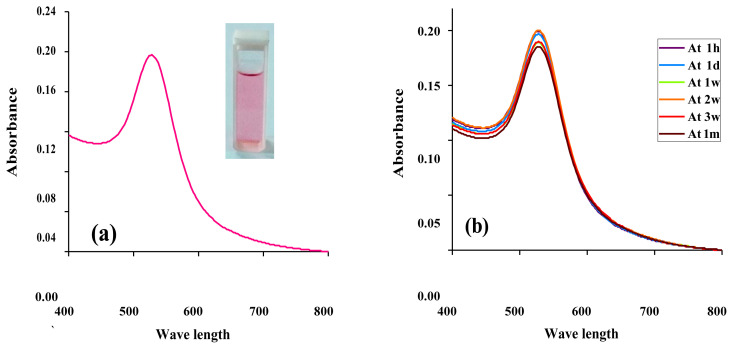
(a) UV-visible spectrum of PUE-AuNPs and (b) stability of PUE-AuNPs at 1 month.

Initially, the synthesis of PUE-AuNPs was confirmed through UV-visible spectroscopy which was confirmed by the characteristic peak of nanoparticles at 528 nm as shown in Figure 2a. The spectrum was taken without any purification or treatment. The stability of nanoparticles is the important factor in the field of application, that is why the synthesized AuNPs were stored at 8 °C over a period of 1 month, and the stability was studied by UV-visible spectrophotometer (Figure 2b).

#### 3.1.1. FTIR spectroscopy of PUE-AuNPs

**Figure 3:**
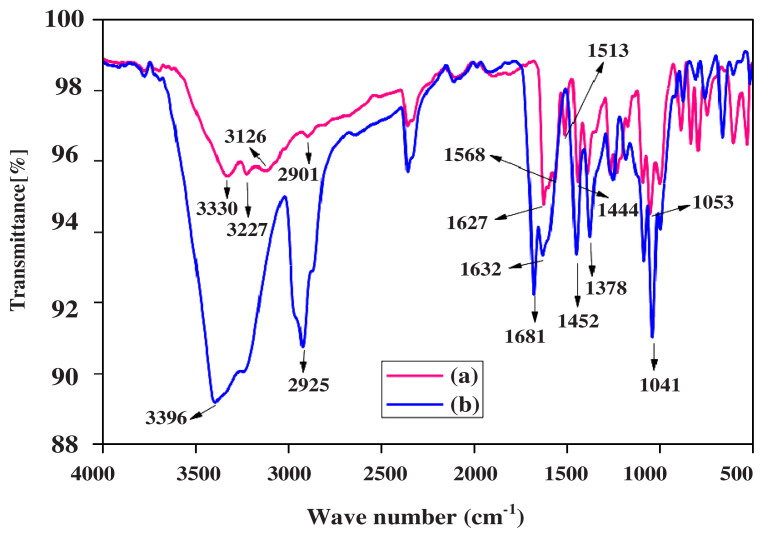
Overlay FTIR spectrum of (a) PUE and (b) PUE-AuNPs.

**Figure 4:**
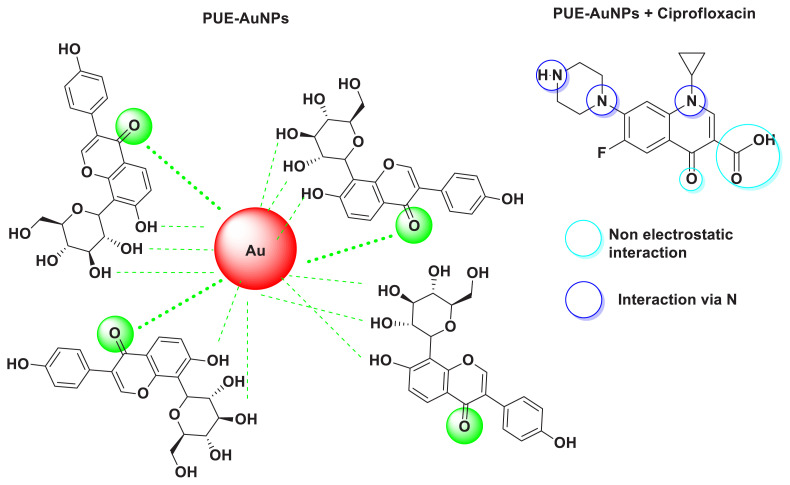
Schematic illustration of the strategy for the synthesis, chemosensing, and probable mechanism of CP-induced aggregation of PUE-AuNPs.

The FTIR analysis identified the functional groups present in puerarin that were responsible for the reduction of Au^+3^ ion to Au^0^. The result showed that hydroxyl (OH), carbonyl groups (C = O), C-O and −C=C present in puerarin were responsible for the AuNPs synthesis. The spectrum of PUE-AuNPs was studied in 4000 to 500 cm^−1^ (Figure 3). The IR spectrum of PUE displayed band at 3330, 3227 cm^−1^ for hydroxyl stretching vibration, 3126 and 2901 attributable to aromatic vibrational C-H, although 1627 confirm the existence of C= O with strong peaks of -C=C stretching vibration around 1568,1513, 1444, and 1053 cm^−1^ (−C-O stretching). Moreover, PUE-AuNPs spectrum displayed peaks in the 3396, 2925, 1681, 1632, 1452, 1378, and 1041 cm^−1^ regions. The sharp peak at 3330 of hydroxyl group became a broad peak of 3396 cm^−1^ and the short peak of aromatic C-H stretching changed from 2901 to 2925 cm^−1^. The peaks also shifted from 1568, 1513, 1444 to 1452 and 1378 cm^−1^. At 1627, carbonyl peak shifted from 1681 to1632 cm^−1^. The stretching of the C-O moved from 1053 cm^−1^ to 1041. These shifting of vibrations in the IR spectrum of PUE-AuNPs as compared to PUE spectrum indicate the contributions of carbonyl, alkene, and polyol groups in the synthesis and stabilization of PUE-AuNPs (Figure 4). Similar observations were also reported in previous literature [25].

#### 3.1.2. Particle size, surface charge, and elemental analysis of PUE-AuNPs

**Figure 5:**
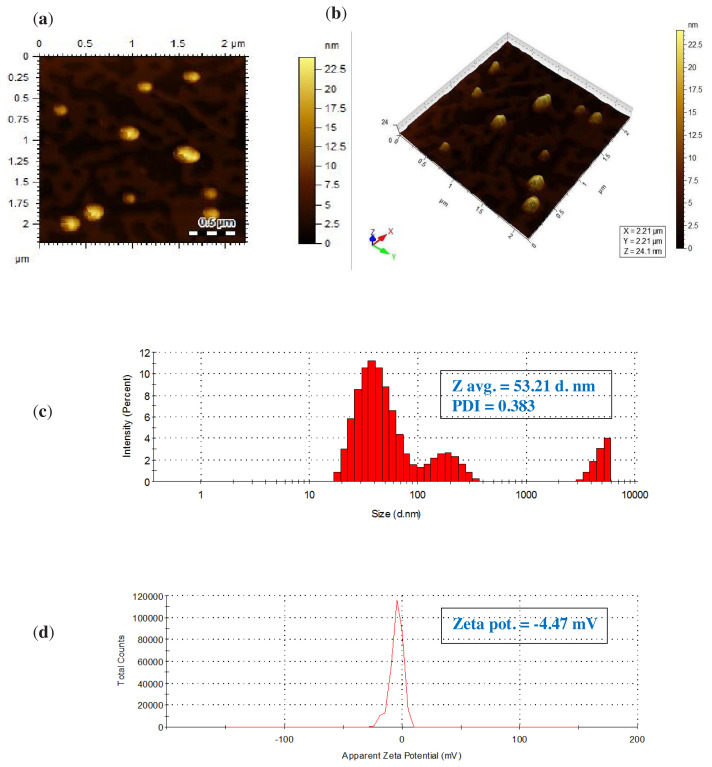
(a) and (b) synthesized PUE-AuNPs AFM micrographs with 3D-view, (c) zeta-sizer with size distribution through intensity, and (d) zeta potential distribution by zeta-sizer.

An atomic force microscopy (AFM) and a zeta-sizer were used for the determination of size and morphology of nanoparticles. AFM images further support the results of SEM by showing sphere-shaped of PUE-AuNPs having size in the range of 19–20 nm (Figures 5a and 5b). A zeta-sizer was used to determine the size, and the PUE-AuNPs size distribution profile had an average diameter of 53.21 nm with polydispersity index (PDI) of 0.383 (Figure 5c). Zeta potential (surface charge) was also used to determine the intensity of nanoparticle interactions with its surroundings. Electrostatic repulsions between particles showed the stability of the particles. The surface charge value of PUE-AuNPs was found to be −4.47 mV (Figure 5d). Similar observations were reported for the silver and gold nanoparticles synthesized from the Polystyrene-block-poly (2-vinylpyridine) [26] and Pyrazinium thioacetate ligand [27].

### 3.2. Application study as a chemosensor

#### 3.2.1. Colorimetric detection of ciprofloxacin with PUE-AuNPs using UV-visible spectroscopy

**Figure 6:**
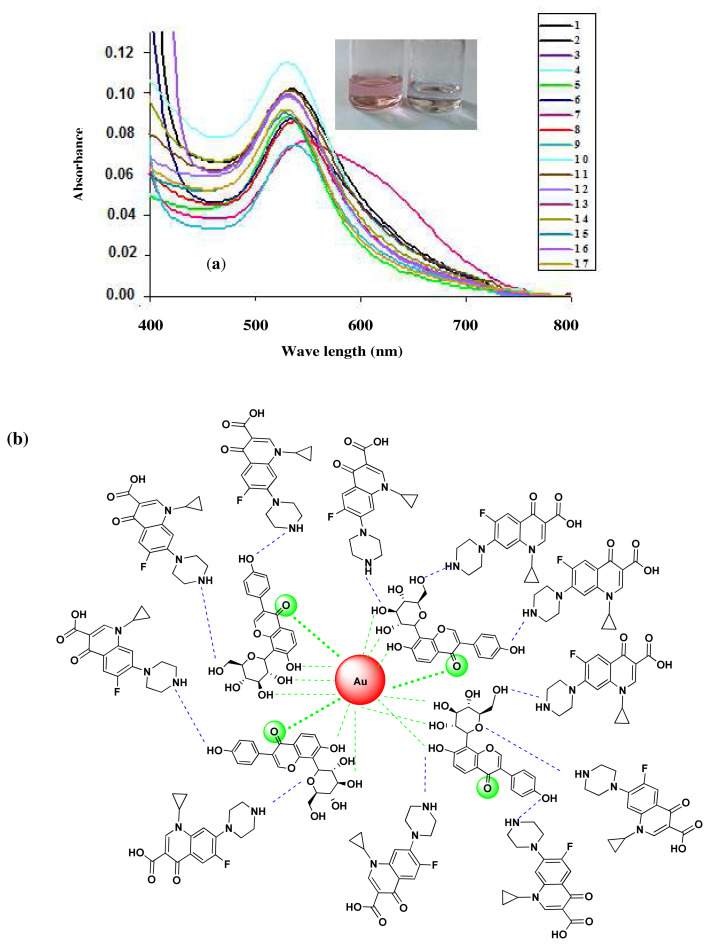

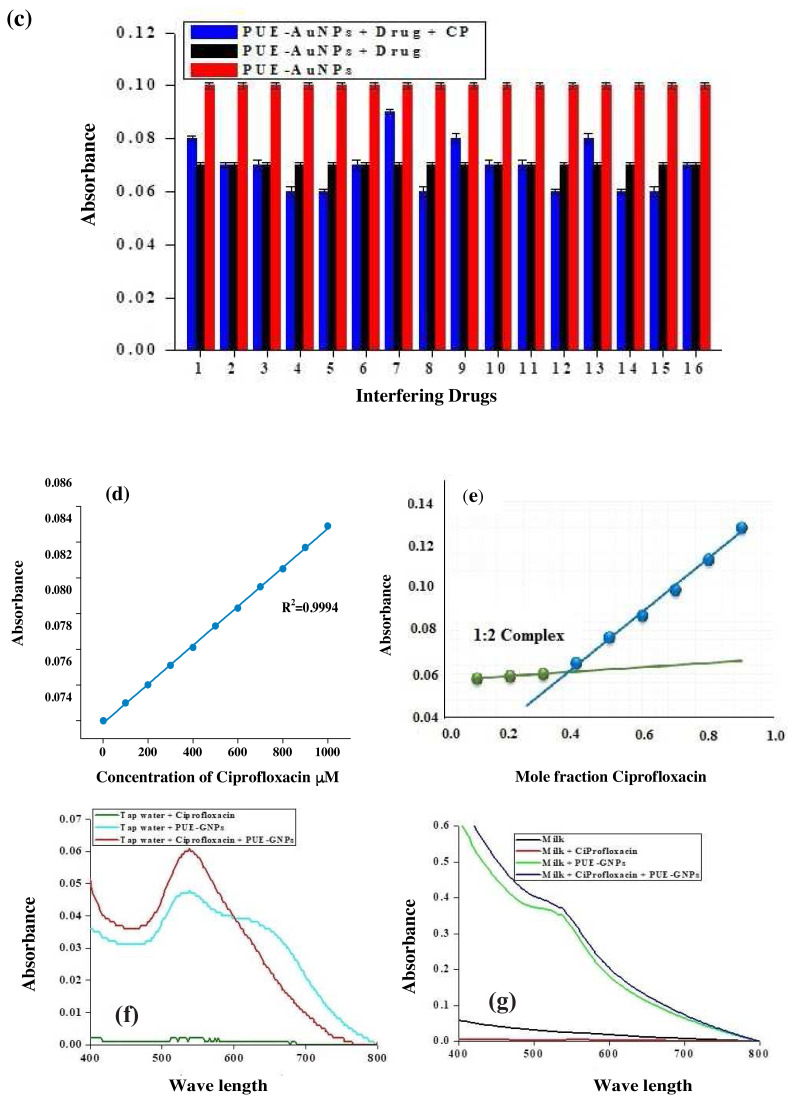
(a) Spectrophotographic study of multiple drugs through using PUE-AuNPs (1= zuclopenthixol, 2= mefenamic acid, 3= paracetamol, 4= flurbiprofen, 5= levetiracetam, 6= levofloxacin, 7= ciprofloxacin, 8= azithromycin, 9= metronidazole, 10= esomeprazole, 11= omeprazole, 12= diclofenac sodium, 13= cephradine, 14= penicillin G. procaine, 15= amoxicillin trihydrate, 16= piroxicam beta cyclodextrin, 17= cefotoxime sodium), (b) complexation between PUE-AuNPs and CP, (c) spectra of UV vis showing the effect of drug interference on CP analysis by PUE-AuNPs (1= zuclopenthixol, 2= mefenamic acid 3=paracetamol, 4= flurbiprofen, 5= levetiracetam, 6= levofloxacin, 7= azithromycin, 8= metronidazole, 9=esomeprazole, 10=omeprazole, 11=diclofenac sodium, 12=cephradine, 13= penicillin G. procaine, 14=amoxicillin trihydrate, 15= piroxicam beta cyclodextrin, 16=cefotoxime sodium), (d) UV-visible spectrum of PUE-AuNPs with varying concentrations (1–1000 μM) of CP, (e) Job’s plot experiment, (f) detection of CP in tap water, and (g) cow milk samples.

In order to evaluate the chemosensing potential the PUE-AuNPs were used with 1mM a series of sixteen drugs solution in 1:1 (v/v) mixture. The solution changed from colorless to pink (Figure 6a). Only CP was detected through spectrophotometric detection method of PUE-AuNPs. It offers a tool for easily detecting CP visually by naked eye. The wavelength change was also recognized at 528 nm to 538 nm by adding CP solution to PUE-AuNPs. These effects that appeared in PUE-AuNPs and CP were due to the changing of binding between the attached groups i.e. hydroxyl (OH) and carbonyl (C = O) of PUE-AuNPs and carboxylic (COOH), carbonyl (C = O) and amino groups of CP (Figure 6a) [26]. Ciprofloxacin’s interaction with PEU-AuNPs is schematically illustrated in (Figure 6b). The remaining analyzed drug solutions did not cause any visible changes in the color and wavelength of PUE-AuNPs (including flurbiprofen, paracetamol, omeprazole, zuclopenthixol, azithromycin, metronidazole, esomeprazole, levofloxacin, mefenamic acid, levetiracetam, diclofenac sodium, cephradine, penicillin G. procaine, amoxicillin trihydrate, piroxicam beta cyclodextrin, cefotaxime and ciprofloxacin) as shown in Figure 6c.

#### 3.2.2. Analytical efficiency of PUE-AuNPs

To examine the analytical efficiency of PUE-AuNPs, several concentration of CP were used to explain the sensitivity in results of PUE-AuNPs based CP sensing. By using UV-visible spectroscopy, numerous CP concentrations were examined against PUE-AuNPs for measuring the sensitive optical response. According to the surface plasmon resonance (SPR) spectrum the absorbance showed strong linearity to the CP concentration in the range of 1 to 1000 μM with the regression constant R^2^ = 0.9994 (Figure 6d). The detection limit (LOD) for CP and the quantification limit (LOQ) are equal to 51 μM and 154 μM, respectively. Moreover, the linear range and LOD of the proposed probe were also compared with other reported methods (Table 1). In addition, Job’s plot experiment could be used to determine the quantitative relationship between the PUE-AuNPs and CP complexes. Different mole ratios of PUE-AuNPs and CP were taken for plotting the spectra by using UV-visible spectrophotometer. The results showed that the stable complex of PUE-AuNPs-CP was formed at 0.3 and 0.7 mole ratios of CP and PUE-AuNPs, respectively (Figure 6e) ideally reported in the literature [28].

#### 3.2.3. Detection of CP in environmental samples

In order to check sensitivity and selectivity of the detector system, practical application was optimized through using a PUE-AuNPs–based nanosensor. We developed the efficacy of CP recognition in environmental samples of tap water taken from Federal Urdu University of Arts Science and Technology, Karachi and cow milk taken from the local market of Karachi. The standard protocol (mentioned in Section 2.5) was used to spike the samples with PUE-AuNPs (1 mM). In tap water (Figure 6f), the enhancement of absorbance was observed when tap water was spiked with PUE-AuNPs and CP, while a broad peak with a shoulder appeared when tap water was spiked in PUE-AuNPs (Table 2a, Figure 7a). However, cow milk sample (Figure 6g) did not show any enhancement and broadening of peak by the addition of PUE-AuNPs and CP (Table 2b, Figure 7b). Such findings indicated the practical use of this detector sensor and we expect that the PUE-AuNPs device proved to be significant in tap water and other environmental samples.

#### 3.2.4. Interaction mechanism of PUE-AuNPs with CP Chemical interaction by FTIR analysis

**Figure 8:**
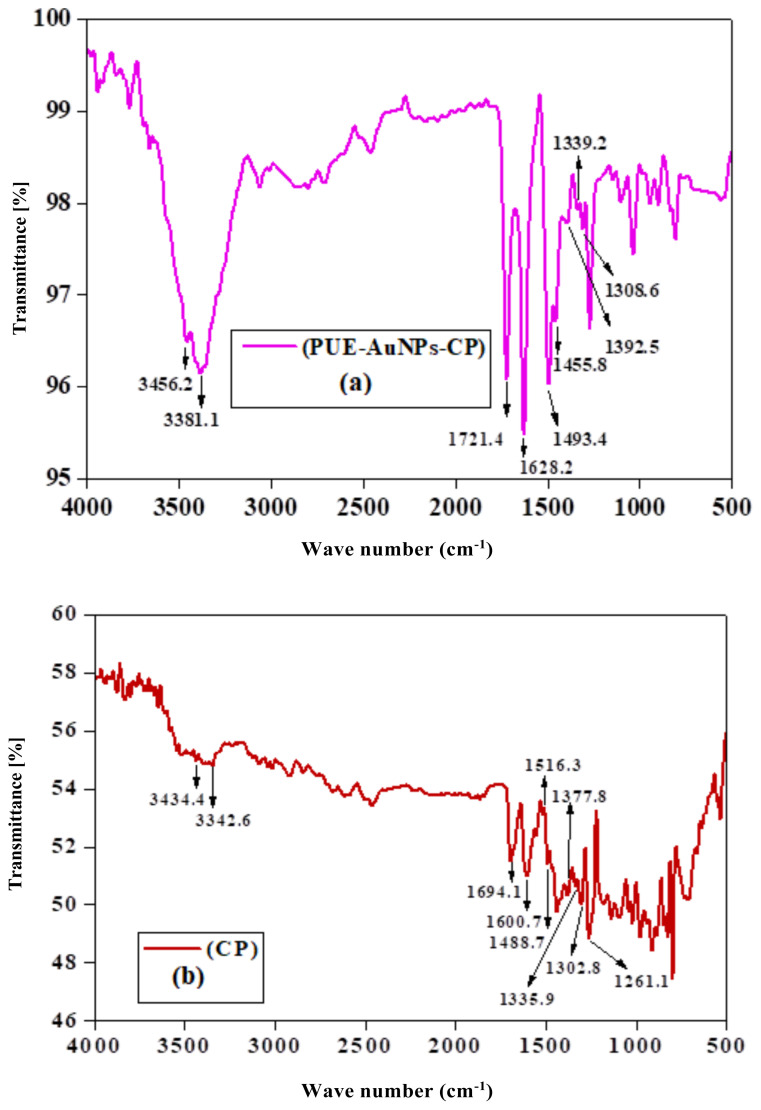

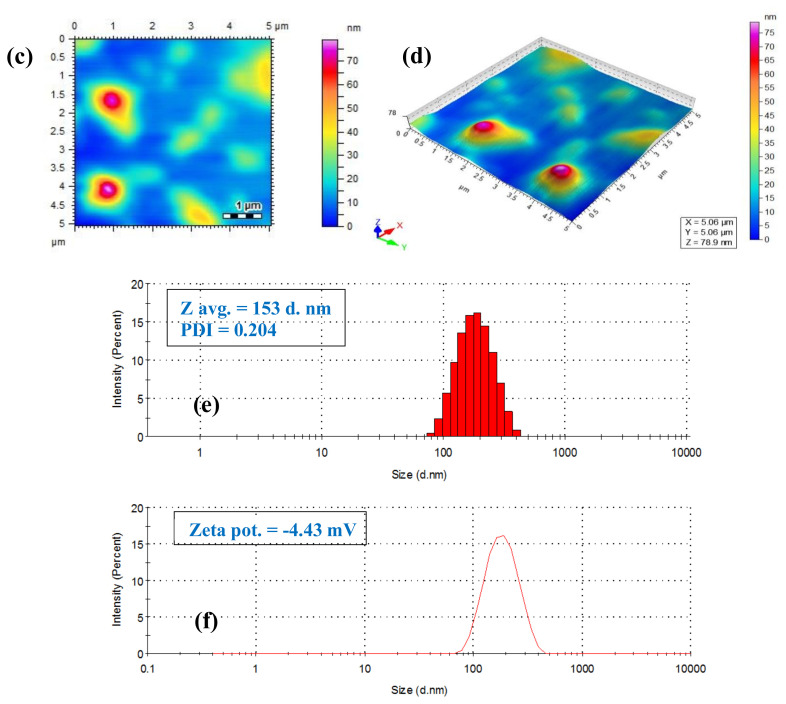
a) FTIR spectrum of PUE-AuNPs-CP complex (b) CP (c and d) size and 3D view micrographs (e) size distribution. (f) surface charge analysis through zeta potential.

The chemical interaction of PUE-AuNPs with CP was studied by FTIR spectroscopy (Figure 8a). The CP-related bands (Figure 8b) occurred at 3434.4, 3342.6, 1694.1 and 1600.7, 1516.3, 1302.8 and 1261.1 cm^−1^ as −NH, −OH vibrational stretching, −COOH (carboxylic), −NH bending vibration, −C=C-, C–N, and C–O, respectively. The peaks have been shifted to 3456.2, 3381.1, 1721.4, 1628.2, 1493.4, 1308.6, and 1270.0 cm^−1^, respectively; in PUE-AuNPs-CP complex (Figure 8a). Enhancement of stretching intensities of −NH and −OH in PUE-AuNPs-CP spectra (Figure 8a) were observed, whereas few peaks appeared to be sharp and dislocated. The obtained results may have suggested that gold nanoparticles (PUE-AuNPs) interacted with OH/NH group of CP, which lead to the crosslinking of two moieties (Figure 4), whereas amine and carboxylic groups might also contribute in nonelectrostatic interaction with the surface of nanoparticles. Our results are very much aligned with previous studies reported in the literature [27].

### 3.3. Molecular recognition of PUE-AuNPs-CP complex via AFM, zeta-sizer, and zeta potential

It was necessary to study the nanoparticles at subnanometric and atomic resolution using the latest atomic force microscopy (AFM) technique. The PUE-AuNPs-CP complex micrographs were screened to evaluate the morphology and dispersity changes that have taken place as it developed a complex with ciprofloxacin. Due to irregular nanoparticles and aggregation, PUE-AuNPs-CP showed a significant shift in size, shape, and dispersity. PUE-AuNPs-CP showed average size of 63–71 nm (Figures 8c and 8d). Dynamic light scattering (DLS) was used to perform zeta-sizer and zeta potential. The CP complex with PUE-AuNPs were distributed at a size of 153 nm and the polydispersity was 0.204 (Figure 8e), while the −4.43 mV was surface charge of PUE-AuNPs-CP complex (Figure 8f). The results are consistent with the previous reported findings [24].

## 4. Conclusion

The present work describes the one pot method for the preparation of puerarin-coated gold nanoparticles (PUE-AuNPs), which was found to be green and nontoxic as compared to previous known traditional methods. It has shown to be an effective quantitative colorimetric protocol for the detection of an antibacterial drug, i.e. ciprofloxacin (CP). The proposed sensor was efficiently used in tap water and cow milk samples for the detection of CP. Detailed characterization of nanoparticles, i.e. size, morphology, as well as interaction of PUE-AuNPs with CP has also been carried out by various spectroscopic and microscopic techniques (AFM, FTIR, UV-visible) and zeta-sizer. In contrast to the presence of other drugs, the PUE-AuNPs–based sensor specifically and selectively detected CP with linear correlation following concentration in the range of 1–1000 μM with a limit of detection of 51 μM. Low cost, easiest preparative method, and excellent selectivity of the proposed ciprofloxacin detector revealed the potential for on the spot alarming process of CP in environmental (tap water and cow milk) samples.

## Figures and Tables

**Figure 7a:**
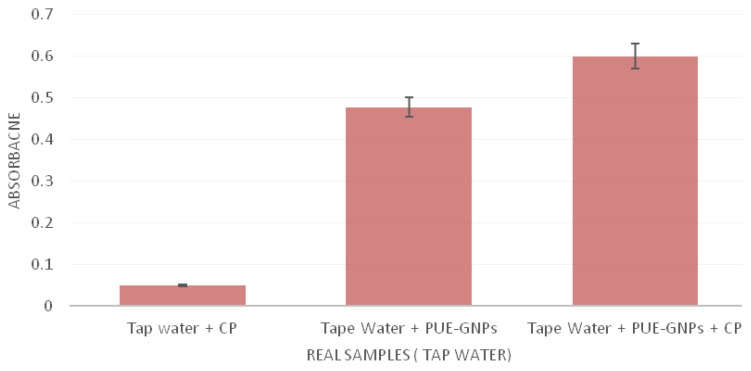
Graphical representation of PUE-AuNPs–based detection of the CP from environmental (tap water) sample.

**Figure 7b:**
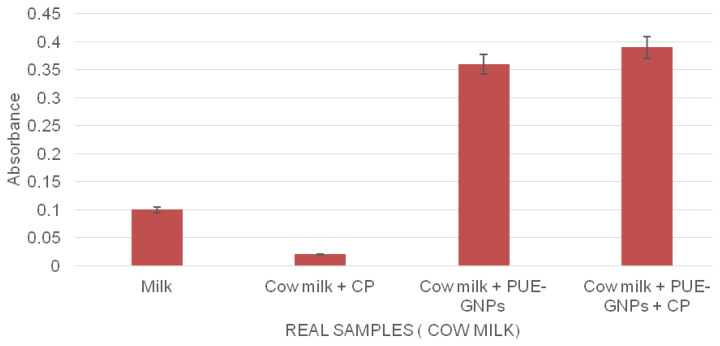
Graphical representation of PUE-AuNPs–based detection of the CP from environmental (cow milk) sample.

**Table 1:** Comparison of the established PUE-AuNPs–based probe for the detection of CP with other previously reported methods.

S. No.	Methods	LOD values	Reference
1	Fluorescence spectroscopy	0.12 μg/mL	[12]
2	Cyclic voltammetry	0.08 μmol L^−^^1^	[13]
3	HPLC with fluorescence	5–1292 ng/L	[14]
4	Colorimetric assay	1 mg/kg	[15]
5	Amperometric and electrophoresis	5.0 mmolL^−^^1^	[16]
6	LCMS Analysis	0.005−2.00 μg kg^−1^	[17]
7	UV-spectroscopy	51 μM	Our study

**Table 2a:** PUE-AuNPs based detection of the CP from environmental (tap water) sample.

S. No.	Real sample (tap water)	Absorbance (nm)
1	Tap water + CP	0.05
2	Tape Water + PUE-AuNPs	0.48
3	Tape Water + PUE-AuNPs + CP	0.60

**Table 2b:** PUE-AuNPs based detection of the CP from environmental (cow milk) sample.

S. No.	Real sample (cow milk)	Absorbance (nm)
1	Cow Milk	0.10
2	Cow milk + CP	0.02
3	Cow milk + PUE-AuNPs	0.36
4	Cow milk + PUE-AuNPs + CP	0.39
